# Elevated transcription of transposable elements is accompanied by het-siRNA-driven *de novo* DNA methylation in grapevine embryogenic callus

**DOI:** 10.1186/s12864-021-07973-9

**Published:** 2021-09-20

**Authors:** Darrell Lizamore, Ross Bicknell, Chris Winefield

**Affiliations:** 1 Bragato Research Institute, Blenheim, Marlborough, New Zealand; 2 Plant and Food Research Ltd, Lincoln, Canterbury, New Zealand; 3 Department Wine, Food and Molecular Biosciences, Lincoln University, Canterbury, New Zealand

**Keywords:** Epigenetics, Transposable element, Embryogenic callus, Het-siRNA, Grapevine, Somaclonal variation, DNA methylation.

## Abstract

**Background:**

Somatic variation is a valuable source of trait diversity in clonally propagated crops. In grapevine, which has been clonally propagated worldwide for centuries, important phenotypes such as white berry colour are the result of genetic changes caused by transposable elements. Additionally, epiallele formation may play a role in determining geo-specific (‘terroir’) differences in grapes and thus ultimately in wine. This genomic plasticity might be co-opted for crop improvement via somatic embryogenesis, but that depends on a species-specific understanding of the epigenetic regulation of transposable element (TE) expression and silencing in these cultures. For this reason, we used whole-genome bisulphite sequencing, mRNA sequencing and small RNA sequencing to study the epigenetic status and expression of TEs in embryogenic callus, in comparison with leaf tissue.

**Results:**

We found that compared with leaf tissue, grapevine embryogenic callus cultures accumulate relatively high genome-wide CHH methylation, particularly across heterochromatic regions. This *de novo* methylation is associated with an abundance of transcripts from highly replicated TE families, as well as corresponding 24 nt heterochromatic siRNAs. Methylation in the TE-specific CHG context was relatively low over TEs located within genes, and the expression of TE loci within genes was highly correlated with the expression of those genes.

**Conclusions:**

This multi-‘omics analysis of grapevine embryogenic callus in comparison with leaf tissues reveals a high level of genome-wide transcription of TEs accompanied by RNA-dependent DNA methylation of these sequences in *trans*. This provides insight into the genomic conditions underlying somaclonal variation and epiallele formation in plants regenerated from embryogenic cultures, which is an important consideration when using these tissues for plant propagation and genetic improvement.

**Supplementary Information:**

The online version contains supplementary material available at 10.1186/s12864-021-07973-9.

## Background

Preferential or obligate outcrossing enables plants to maintain genetic diversity in wild populations [1], and is common among perennial plants, which employ a range of physiological and genetic strategies to achieve self-incompatibility [2]. As a result, the genomes of these species are highly heterozygous. But efficient cultivation of these species requires homogenous populations in which preferential phenotypes are fixed. The centuries-old solution to domesticating highly heterozygous species is clonal propagation.

Modern grapevine (*Vitis vinifera* subsp. *vinifera*) is presumed to have been derived from the wild European grapevine (*Vitis vinifera* subsp. *sylvestris*) around 8,000 years ago [3]. The long history of grapevine cultivation provides an unique example of vegetative propagation. Pliny the Elder described the clonal cultivation of grapevine nearly two millennia ago in *Natural History* (AD 77). Today, the names of many centuries-old varieties such a as ‘Pinot noir’ and ‘Chardonnay’ are well known to wine consumers [4]. They provide signals of quality by which wine is marketed, but can also represent an obstacle to grapevine breeding efforts, which produce new and unknown varieties [5, 6].

However, plant genomes are not invariable in the absence of sexual reproduction. Occasionally, mutations in somatic tissues become fixed when material is collected from chimeric plants for propagation. Where mutations produce visible phenotypes, termed ‘bud sports’, they can provide genetic variation from which to select valuable traits, thus providing an alternative route for crop improvement. Seedless varieties such as ‘Sultana’ and white-skinned varieties such as ‘Pinot blanc’ are examples of economically important traits that have become established through the artificial selection and clonal propagation of somatic mutations that would be under negative selective pressure in the wild [7].

Sequence comparisons show that most variation among grapevine clones is due to the activity of transposable elements (TEs) [8]. These repeat sequences are ubiquitous in eukaryote genomes and comprise the majority of the nuclear DNA in many plant species. They are broadly categorised into Class I elements (retrotransposons), which replicate via an RNA intermediate, and Class II elements (DNA transposons), which mobilise via the excision and re-insertion of a section of double-stranded DNA within the host genome [9]. Because of these differences in mobility, retrotransposons tend to increase in abundance within the host genome and typically outnumber DNA transposons. Analyses of the grapevine reference genome show that TEs account for approximately half of the nuclear DNA including representatives of eight out of the nine recognised TE superfamilies (the exception being *Tc1-Mariner*) [10, 11].

Because of their potential mutagenicity, host species have evolved ways to repress TE activity by transcriptional gene silencing (TGS) and post-transcriptional gene silencing (PTGS) [12]. The large complement of silenced TEs in plant genomes acts as a source of sequence homology by which active TEs can be recognised and targeted for TGS, in a process known as RNA-directed DNA methylation (RdDM). In the canonical RdDM pathway, the plant-specific RNA polymerase RNA Pol IV transcribes heterochromatic TEs associated with H3 histones methylated at lysine 9 (H3K9me). The transcripts are converted to double-stranded RNA by RNA-DEPENDENT RNA POLYMERASE 2 (*RDR2*) and processed into 24-nt small interfering RNAs (siRNAs) by DICER-LIKE 3 (*DCL3*). These so-called heterochromatic siRNAs (het-siRNAs) direct ARGONAUTE 4 & 6 (*AGO4* & *AGO6*) to nascent TE transcripts generated by RNA Pol V in a homology-dependent way, where they trigger the methylation of cytosine bases and ultimately H3K9me. In cases where TEs escape TGS (either through the introduction of new TEs for which no homology exists in the genome, or failure of the RdDM pathway), the host cell needs to both initiate PTGS to remove TE transcripts, and establish *de novo* methylation of the TE locus. The PTGS and non-canonical RdDM pathways depend on 21–22 nt siRNAs from a variety of sources including Pol II TE transcripts converted to dsRNA by RDR6 and subsequently processed into 21–22 nt siRNAs by *DCL2* & *DCL4*. These secondary siRNAs target TE transcripts for degradation via RNA interference (RNAi) and guide homology-dependent TE methylation in a non-canonical RdDM pathway known as RDR6-RdDM [12, 13].

Without maintenance, cytosine methylation that represses TE transcription would be passively lost at each replication cycle. To prevent this, cytosines in symmetrical CG and CHG motifs (where H is C, A or T) are actively copied to the newly synthesised DNA strand by METHYLTRANSFERASE 1 (*MET1*) & CHROMOMETHYLASE 3 (*CMT3*) respectively. Cytosine methylation in the asymmetrical CHH context cannot be copied between strands. Instead it is applied at each cellular generation by *CMT2*, which methylates heterochromatic DNA, or through RdDM via *DRM2* [14].

Mobilisation of TEs can alter genes and their expression in subtle and complex ways. Besides disrupting open reading frames and protein recognition sequences, transposition events can cause gene duplication, alternative splicing and create new regulatory networks [15, 16]. Host efforts to epigenetically repress TE expression can affect nearby DNA, and indeed have been co-opted for gene regulation in some cases [17, 18]. Similarly, many TEs have accumulated stress-response regulatory elements over time. As this upregulates their activity in response to environmental stress, such TEs may even provide the host with a mechanism for increasing variation at times when it is most needed [19]. Therefore, besides their role as genomic ‘parasites’, domesticated TEs occasionally also provide a useful mechanism for adaptation that can be co-opted for crop improvement [20, 21].

Through judicious application of phytohormone regimes during tissue culture, certain somatic plant tissues can be converted to totipotent embryogenic callus (EC). The de-differentiated cells of EC can then be either propagated or allowed to differentiate into bipolar embryos. Somatic embryogenesis has been used to study the biological process of embryogenesis and to clonally propagate certain crops, such as forestry species [22]. In many species, including grape, embryogenic callus is also a target tissue research involving gene discovery, transgenic techniques and gene editing using CRISPR/Cas-based approaches, as it minimises chimerism in regenerated plants [23–25]. But tissue culture is known to exert a mutagenic effect on plant cell lineages, leading to genomic changes known collectively as somaclonal variation. Certain plant TEs are activated by cell culture, and their mutagenic potential may be exacerbated by the abiotic stresses and phytohormones that are part of the process [26, 27]. Plants, including grapevine, regenerated from EC have also been seen to harbour epigenetic DNA changes [28–31]. Epiallele variation of this type may be particularly relevant to vegetatively propagated crops, though epialleles have also been seen to persist for multiple seedling generations [32]. In fact, stable epigenetic variation has been used to differentiate grapevine clones [33] and may be a key factor underlying the ‘terroir’ differences observed between identical clones in different winegrowing regions [34, 35].

Somaclonal variation has certain advantages for perennial crop improvement. Unlike hybrid varieties, novel clones can be deployed by the industry with no effect on varietal identity or the need for multi-generation backcrossing. But for somaclonal mutation to be used efficiently, a species-specific understanding of the types and rates of genetic and epigenetic change induced by somatic embryogenesis is needed [36]. We aimed to study the mechanisms of genetic and epigenic somaclonal variation in EC by comparing these cultures with leaf tissue, which is terminally differentiated. To do this we used whole-genome bisulphite sequencing to map the methylomes of EC and leaf tissue at single base-pair resolution. From these we analysed how the patterns of genome-wide methylation in these tissues differ across genes and TEs, and in particular at loci where these two feature types overlap. To understand how the expression of TEs and their genic context is related to their epigenetic state, we then compared the differential expression of TEs and the genes with which they are co-located. Finally, we analysed the small RNA complement of each tissue, since these molecules guide homology-dependent TE silencing. This multi-‘omics approach provides a snapshot of the activity and impact of TE expression and silencing in EC, contrasted with leaf tissue, which is frequently used to compare methylation between and within plant species.

These results, and future work to understand the impact of these events on regenerated plants, will have practical implications for the use of EC for somaclonal crop improvement. Furthermore, observations of the genomic effects of this TE activity and host response in totipotent cells *in vitro* may also help clarify the role of developmental relaxation of TE silencing (DRTS) in whole plants.

## Results

### TE methylation in Embryogenic callus

To compare the genome-wide DNA methylation profiles of EC with those of leaf tissue, whole-genome bisulphite sequencing was performed for both tissue types. Bisulphite conversion rates were 99.59 and 99.72 % for leaf and EC libraries respectively, and global read coverage after mapping was 36-fold for the leaf library and 32-fold for the EC library (Suppl. Table S1).

**Fig. 1:**
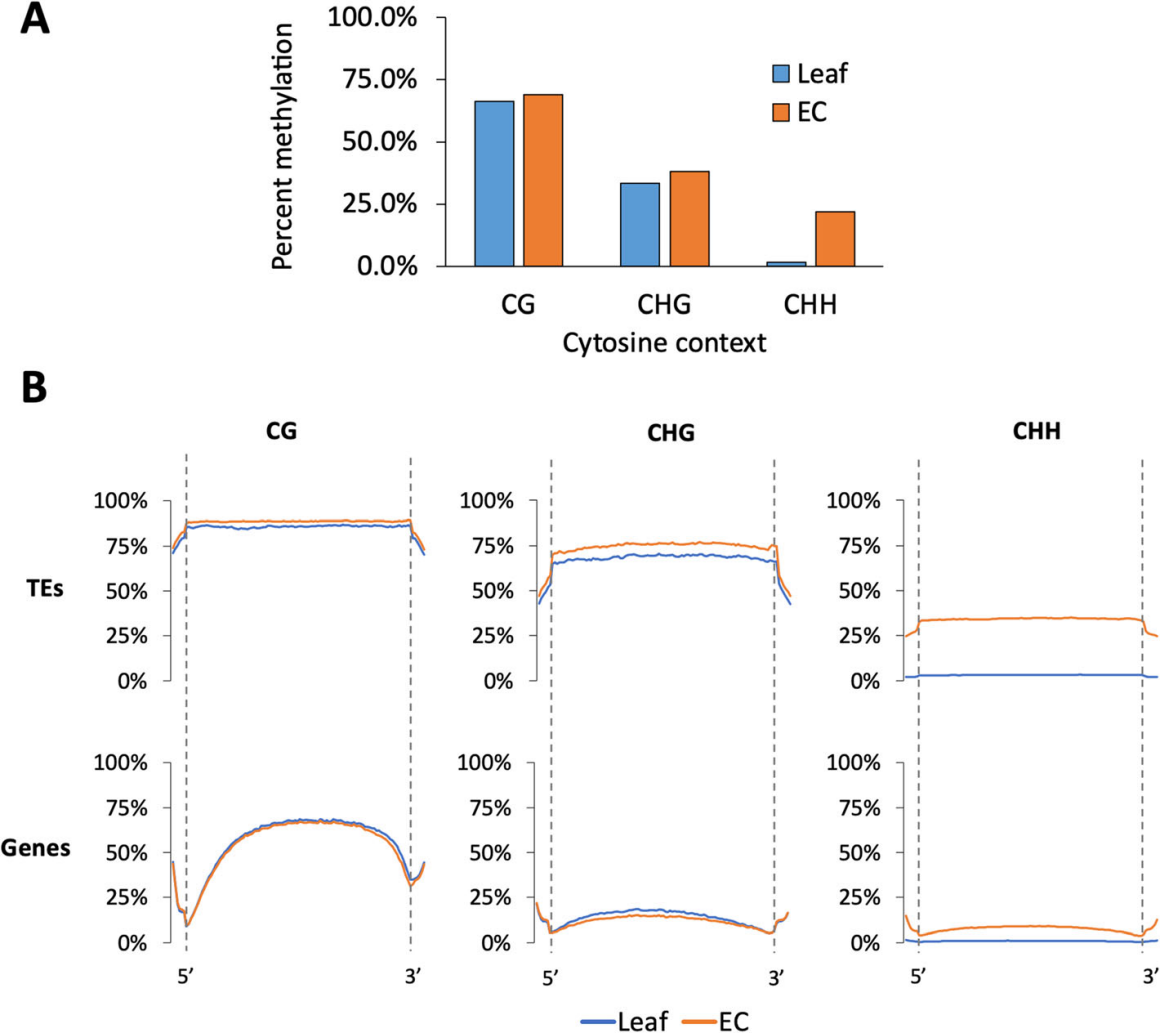
Cytosine methylation differences between leaf and EC. **A**: Global methylation by cytosine context for both tissue types. **B**: Profiles of DNA methylation across TE and gene feature types by cytosine context, with adjacent 1 kb flanking regions shown. Dashed lines indicate feature boundaries

Cytosine methylation in EC was found to be higher than in leaf for each cytosine context (Fig. 1A). This is consistent with previous reports that have shown genome-wide dispersed hypermethylation in callus cultures of *Arabidopsis*, maize and rice [37–39]. Most notable was the relatively high proportion (21.8 %) of CHH methylation in EC, which was very low (1.7 %) in the leaf tissue samples. To further study the genomic context of DNA hypermethylation in EC, we analysed methylation across TEs and genes separately. This required that we first annotate repeat loci in the reference genome based on sequence similarity with published *Vitis vinifera* repeat sequences, which yielded 222,411 annotations (Suppl. Fig. S1; the annotation track is available for download at DOI: 10.6084/m9.figshare.14709816).

In both tissue types, genes were more highly methylated than adjacent DNA in the CG context but less methylated than adjacent DNA in the CHG context (Fig. 1B). In contrast, regardless of tissue type or cytosine context, methylation across TEs was found to be higher than that of adjacent DNA. For CG and CHG contexts, methylation in EC was higher than that of leaf tissue across annotated TEs, but not across genes, indicating that the overall differences in global methylation percentages were associated with hypermethylation of TEs in the callus (Fig. 1B). The exception to this trend was CHH methylation. Although CHH methylation in EC was particularly high across TEs, CHH methylation across genes was also higher in EC than in leaves. A potential explanation for this is the presence of TEs containing hypermethylated CHH sites situated within genes. To determine whether this was indeed the case, we further analysed TEs that are co-located with genes (defined as those that overlapped gene annotations by at least 1 bp), which we termed ‘genic TEs’, and ‘intergenic TEs’ (those that do not overlap gene annotations) separately. By overlaying the TE and gene feature annotation sets, we found that 49,257 (21.9 %) of TEs overlapped annotated genes. Of the 31,845 gene features in the V 2.1 annotation set, 11,311 (35.6 %) were co-located with TEs. This indicates that in grapevine many TEs are dispersed through protein-coding regions of the genome.

**Fig. 2:**
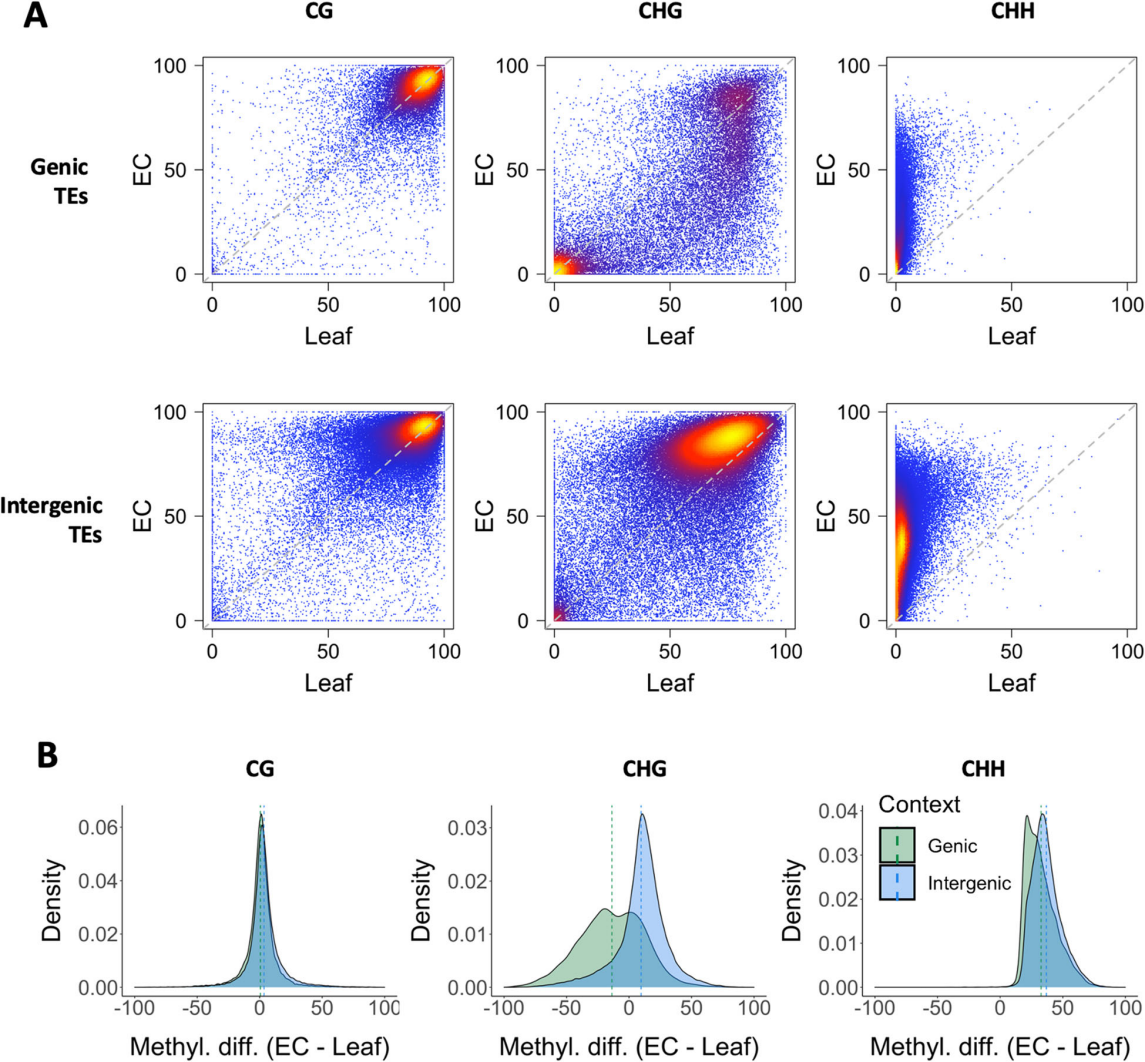
Comparison of TE methylation between EC and leaf. **A**: Density-coloured scatter plots show the percentage methylation for each TE feature in leaf vs. EC tissue across three cytosine contexts (CG, CHG and CHH). TEs co-locating with genes (genic TEs) are plotted separately to intergenic TEs for comparison. **B**: Density plots of fractional TE methylation differences between EC and leaf tissue, per cytosine context. Only features with at least 20 % methylation in at least one tissue type are included. Dotted lines indicate the mean methylation difference; density is in arbitrary units

Methylation in the CG context was found to be constitutively high across both genic TEs and intergenic TEs in leaf tissue and EC (Fig. 2A). However, while intergenic TEs generally had high GHC methylation in both tissue types, genic TEs showed a bimodal distribution, particularly in leaf tissue where TEs co-locating with genes were either highly CHG methylated or relatively unmethylated in this context. Interestingly, many genic TEs that had high CHG methylation in leaf tissue showed reduced methylation in EC. CHG methylation acts as a functional signal for TE silencing in plants, and loss of this signal can permit TE expression to disrupt nearby genes. This has been seen in the case of the MANTLED locus in oil palm, which is the site of a TE insertion related to the *Karma* rice retrotransposon in the intron of the homeotic gene DEFICIENS. Loss of CHG methylation at this locus leads to alternative splicing, resulting in a prematurely terminated gene transcript [31]. While a proportion of genic TEs demonstrated CHG hypomethylation in EC relative to leaves, intergenic TEs tended to show slightly higher CHG methylation in EC than in leaf tissue.

Asymmetric CHH methylation, which is indicative of active *de novo* methylation, was largely absent in leaf tissue. In contrast, TEs in EC displayed significant CHH methylation, particularly across intergenic TEs (Fig. 2A). To better visualise the degree to which TEs varied in methylation between the two tissues, density plots were generated for all TEs with at least 20 % methylation in one tissue (Fig. 2B). While CG methylation of TEs appeared to be independent of co-located genes, intergenic TEs had higher CHG methylation in EC, whereas a large proportion of genic TEs showed lower CHG methylation. As described above, CHH methylation of TEs was higher in EC, with the effect greatest for intergenic TEs.

### High TE expression in EC

Since methylation in plants is functionally important for silencing TEs, we wanted to see if the observed genome-wide changes to TE methylation were associated with a corresponding difference in TE expression. To do this, we performed sequencing on triplicate RNA libraries from each tissue type. The two genes with the highest read counts in leaf tissue were VIT_213s0019g0263 (mean 63,259 reads per million mapped) and VIT_207s0031g03000 (mean 8,480 reads per million mapped). Closer inspection revealed sequence homology with chloroplast genes *psbA* (photosystem II D1 protein) and *rbcL* (RuBISCO large subunit). This is likely due to over-assembly of the reference genome, as has been reported elsewhere [40]. Read counts for these two genes were removed from count tables prior to normalisation, in order to compare the expression of nuclear genes between the two tissues.

**Fig. 3:**
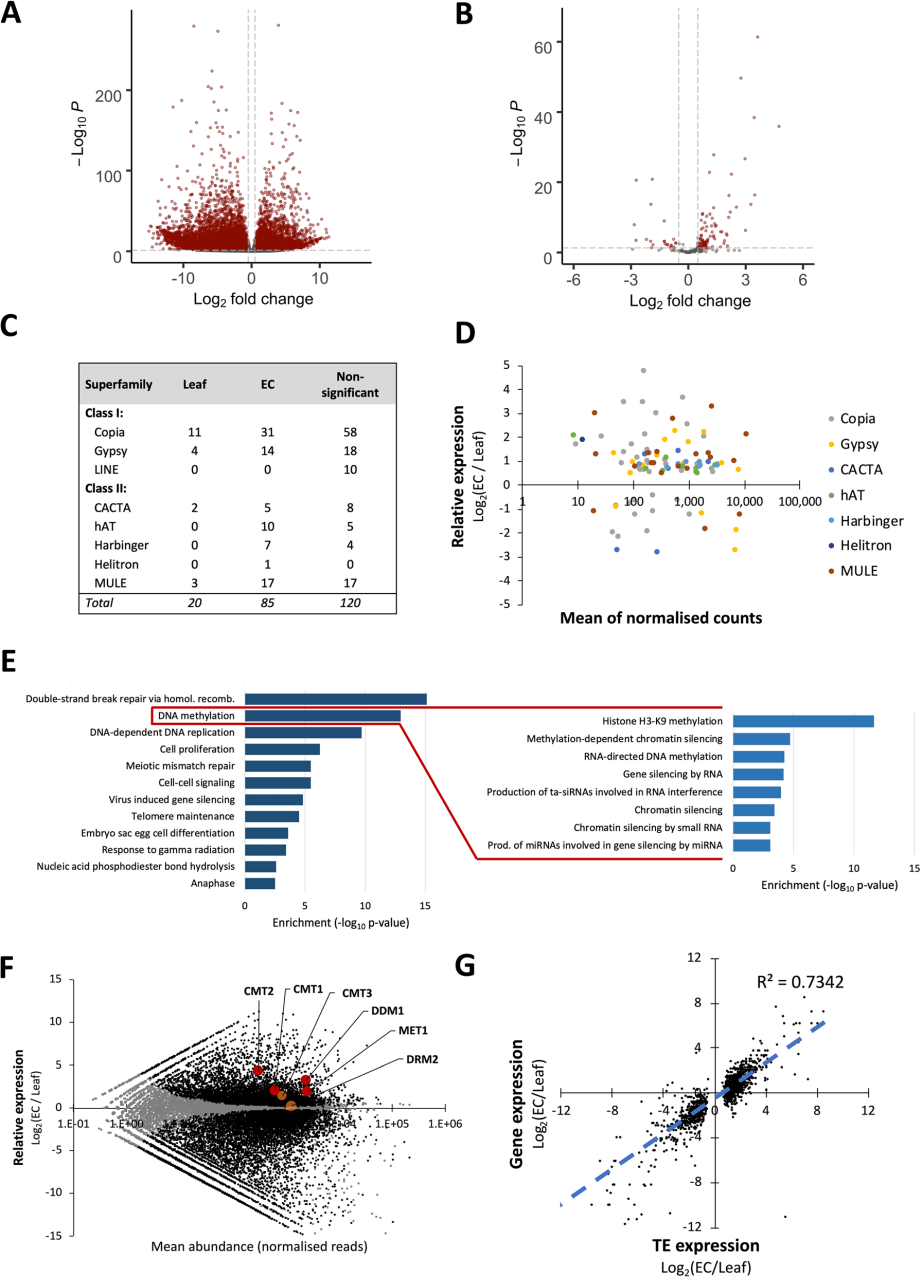
Transcription of TEs in grapevine EC, compared with leaf. Volcano plot of gene differential expression analysis for **A**: Genes and **B**: TEs. Features significantly DE (*p* < 0.05) with |Log2 Fold Change (EC/Leaf)| > 2 are highlighted in red. **C**: Counts of TE families with relatively higher expression levels in each tissue type. **D**: m-a plot of TE families differing significantly in expression between leaf & EC tissue, coloured by TE superfamily. **E**: GO terms enriched among DE genes highly expressed in EC relative to leaf, grouped by similarity. Enriched terms grouping under “DNA methylation” are shown in the expansion to the right. **F**: m-a plot of gene expression (expression in EC / expression in leaf ). Genes not passing the significance threshold (adjusted *p* < 0.05) are shown in grey. DNA methyltransferase genes are highlighted in red where significantly DE and in orange where no significant difference is observed. **G**: Relative expression of DE TEs between tissue types, compared with the relative expression of co-located DE genes

We found that the proportion of reads mapping to TEs was significantly higher (*p* < 0.001) in RNA libraries of EC (9,278 ± 508 reads per million), compared with those from leaf (3,820 ± 735 reads per million). Differential expression analysis identified 14,229 differentially expressed (DE) genes, with 8,210 more highly expressed in leaf and 6,019 more highly expressed in EC (Fig. 3A; Suppl. Table S2). Of the 105 differentially expressed TE families identified, 85 were more highly expressed in EC (Fig. 3B C; Suppl. Table S3). Each of the transposon superfamilies, with the exception of LINEs, included TE families more highly expressed in EC compared with leaf tissue (Fig. 3C & D). Although differential expression of LINE elements did not cross the threshold for significance, all LINE families had higher mean expression among EC replicates compared with leaf replicates (Suppl. Table S3).

Genes more highly expressed in EC were summarised into 12 ontological categories by enrichment analysis. These categories showed an overall upregulation of genes associated with the functions of DNA replication and cell division (Fig. 3E). Grouped within the second most enriched category, ‘DNA Methylation’ (GO:0006306), were 8 other significantly enriched GO terms associated with epigenetic activity (Fig. 3E, inset) These ontologies cover the breadth of small-RNA driven chromatin modification and DNA methylation described above, including RdDM. Genes more highly expressed in leaf grouped into 36 ontological categories, including terpenoid production, defence response and photosynthesis (Suppl. Fig. S2). To further determine how the observed hypermethylation of DNA in EC relative to leaf might relate to gene expression, we compared the expression of DNA methyltransferase genes previously described in grapevine [41]. Of these, *DDM1*, *CMT1*, *CMT2* and *MET1* were found to be more highly expressed in EC, while DRM2 and CMT3 showed no significant difference (Fig. 3F).

Since TEs exist as multiple copies in individual genomes, short sequencing reads frequently map to multiple genic loci. In order to quantify TE transcript abundance, bioinformatic tools such as TEtranscripts count ambiguously mapped reads to compare the expression of TEs at the family level, rather than individual insertions. Given the significant differences between the methylation profiles of genic and intergenic TEs, we wanted to know whether there was any relationship between the expression of specific TE loci and the transcription of co-located genes. To determine this, DE analysis was subsequently performed using only uniquely mapping reads. Of the 2,278 discrete genic TE loci (TE loci overlapping annotated genes) that passed the significance threshold for differential expression using this approach, 1,576 (69 %) were co-located with genes also passing the threshold for significant DE. Expression of TEs was closely correlated with that of co-located genes, providing evidence that in general the transcription levels of individual TEs are linked to the level of transcription of the genes within which they are located (Fig. 3G).

### Heterochromatic siRNAs accumulate in EC

Sequence-specific recognition of TEs for RdDM and PTGS depends on the presence of small RNAs to guide protein complexes to their targets. Our comparison of the DNA methylation profile and transcriptome of grapevine EC with leaf tissue demonstrated a marked difference with regards to TE silencing across the genome. To interrogate the dynamics of the host cell’s TE repression in these tissues we decided to sequence the sRNA complement present.

After adapter trimming and quality and fragment length filtering, 85 and 69 % of the reads remained for the leaf and EC small sequencing libraries respectively (Suppl. Table S4). Micro RNAs (miRNAs) accounted for 2,040,584 reads in the leaf library (25.9 % of total reads), but only 27,896 reads (0.4 %) of the EC library. Of the miRNA expressed in leaf tissue, 33.3 % were from a single miRNA family, miR166, as previously reported [42]. To compare the abundance of siRNAs apart from this difference in miRNA expression, siRNA were normalised to the size of read libraries after miRNA removal.

**Fig. 4:**
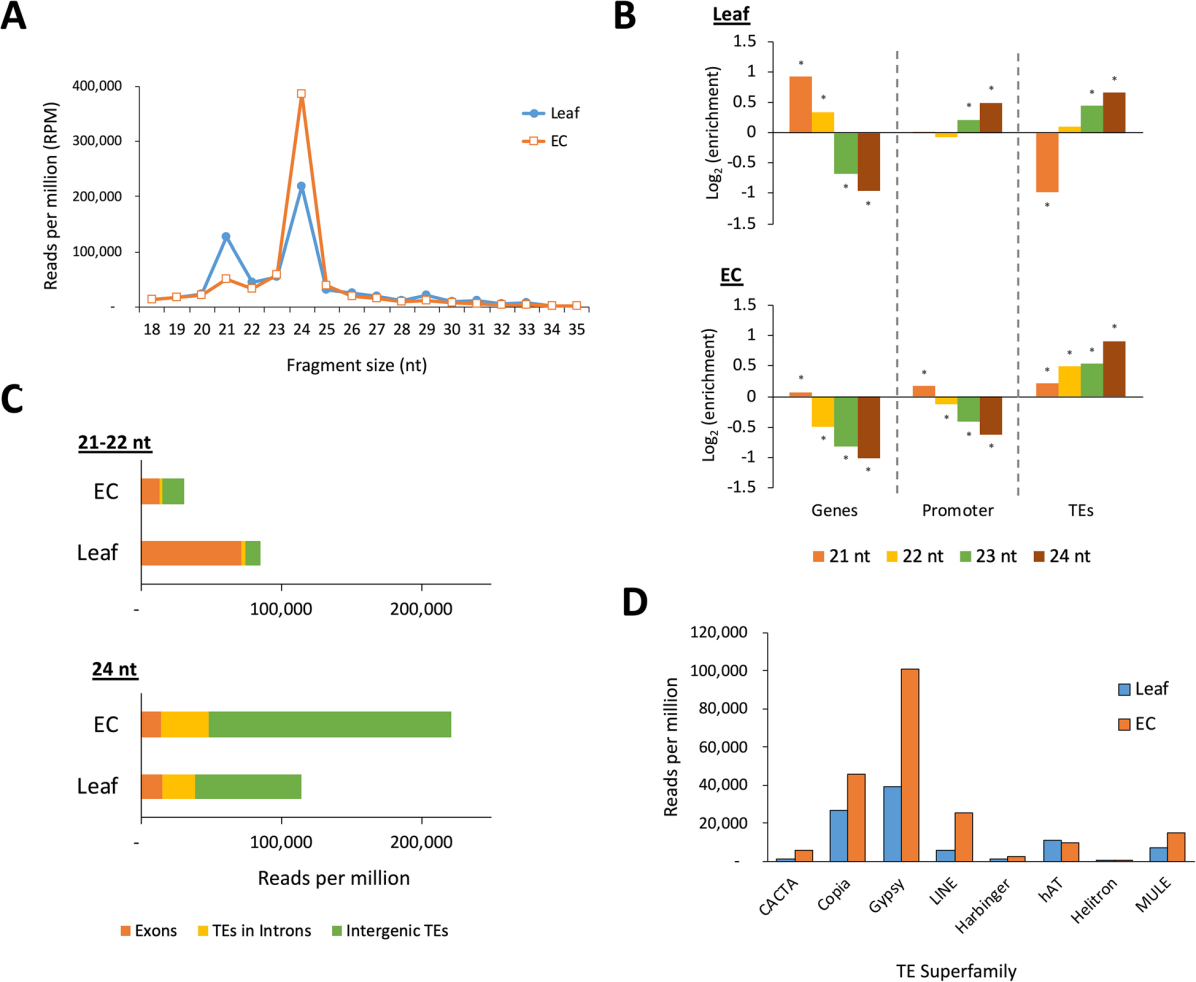
Small RNA abundance in leaf and EC tissue **A**: Size distribution of siRNAs in each tissue type in reads per million (RPM) after miRNA filtering. **B**: Enrichment analysis of siRNA DicerCall clusters across genomic features (* indicates significance at *p* < 0.01). **C**: Feature mapping for siRNAs by size category, normalised as RPM. **D**: Counts of 24 nt siRNA reads mapping to each TE superfamily, for each tissue type (in RPM)

After removing miRNAs, 21 nt siRNAs, which are associated with both non-canonical RdDM (TGS) and RNAi (PTGS), were found to be higher in leaf tissue (Fig. 4A). In contrast, 24 nt het-siRNAs, which initiate canonical RdDM, were higher in EC. The mapped siRNA were clustered by ShortStack into a total of 89,597 DicerCall clusters in leaf and 227,847 DicerCall clusters in EC. To determine the location bias of the clusters, GAT was used to asses enrichment across three feature types: genes, promotors (regions 2 kb upstream of genes) and TEs. In leaf tissue, 21 nt siRNA clusters were found to be enriched in genes and depleted in TEs, whereas 23 and 24 nt siRNA clusters were depleted in genes and enriched in promoters and TEs (Fig. 4B). EC showed depletion of siRNA clusters of all sizes except 21 nt in genes and their promoters but an enrichment for clusters of all siRNA sizes in TEs, with 24 nt siRNAs most enriched.

For 21–22 nt siRNAs, which include secondary and phased small RNAs, the relatively higher abundance in leaf was due to sequences associated with gene exons (Fig. 4C). However, the increase in 24 nt siRNAs in EC was particularly associated with TEs, mostly in intergenic space. As in the case of the mRNA transcript libraries, the relative abundance of 24 nt sRNAs was not limited to individual TE superfamilies. Rather, the increase in het-siRNAs was associated with Gypsy, Copia, LINE, CACTA & MULE superfamilies, with those mapping to Gypsy elements accounting for 56.2 % of the difference in 24 nt siRNAs between the two tissue types (Fig. 4D).

**Fig. 5:**
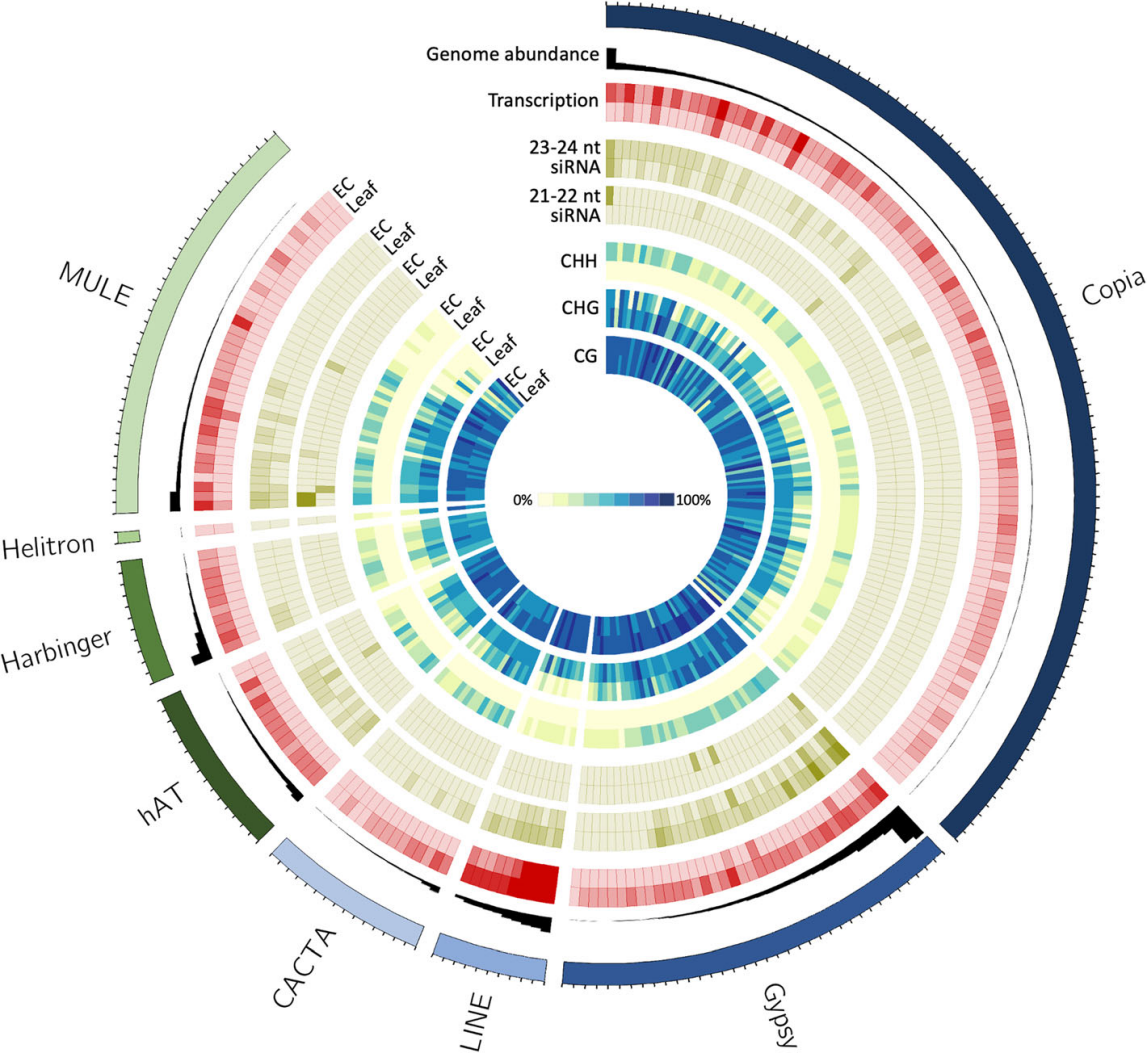
Transcription, small RNA abundance and methylation in embryogenic callus and leaf by TE family. Mean data is reported per TE family, which are represented by outer tick marks, grouped by superfamily and sorted in order of repeat abundance in the *Vitis vinifera* genome. Tracks, starting from the outside are: *Vitis vinifera* TE families, grouped by superfamily (ideograms); number of copies per genome (black histogram); normalised transcript abundance (red heat maps); normalised 21–22 nt siRNA abundance (outer brown heat maps); normalised 21–22 nt siRNA abundance (inner brown heat maps); mean CHH methylation, mean CHG methylation and mean CG methylation (yellow to blue heat maps, see scale in the centre)

Integrating all three datasets showed that in EC the increased TE expression, siRNA abundance and CHH methylation relative to leaf are associated with the same TE families (Fig. 5). Furthermore, the TE families that were more abundant in the grapevine genome appeared to be those for which the expression and *de novo* silencing responses were highest.

## Discussion

Tissue culture, including somatic embryogenesis, has long been recognised to have a mutagenic effect on plant genomes that is mediated by TE activity [43–46]. This fact can frustrate efforts to recover specific genotypes following tissue culture [47, 48]. Tang and colleagues, for example, found that somaclonal mutations were far more abundant than off-target mutations in genome-edited rice lines [49]. But TE-derived somaclonal variation has the potential to provide valuable diversity [50]. In rice the *Copia*-type retrotransposon *Tos17*, which is stimulated by prolonged tissue culture and has been shown to preferentially insert into gene-rich regions, has been used to produce a population of over 47,000 insertion mutants [51]. Similarly, tissue culture induced transposition of *LORE1* has been used for saturation mutagenesis in *Lotus japonicus* [52]. This diversity is particularly attractive as a source material from which to make selections when other crop improvement techniques are limited. In the case of grapevine, for example, the fact that varietal identity is a premium indicator of wine quality means that support for breeding approaches is limited for commercial reasons.

Since Barbara McClintock’s discovery of “dissociation” (*Ds*) elements in maize [53], most studies investigating the role of transposition in somatic mutagenesis have focussed on individual TE families. This is because most active transposons were identified and characterised following the observation of an atypical phenotype. However, the advent of massively-parallel sequencing techniques, together with publicly available reference genome assemblies, enable the expression and silencing of the entire mobilome (mobile portion of the genome) to be studied simultaneously. To do this, we used three sequencing approaches to characterise the methylation, transcription and small RNA-targeting of TEs in EC cultures. We compared these with leaf tissue, a terminally differentiated tissue frequently used as a reference for studies comparing the methylation of different plant tissues and species [39, 54–57].

The genome-wide methylation levels observed in leaf samples were comparable to those that have been previously reported for *Vitis vinifera* [55]. The most distinctive difference between the epigenetic state of EC and leaf tissue was the relative abundance of CHH methylation in EC cells, which was almost absent in leave tissue. Increased CHH methylation has been previously reported in totipotent cell cultures of rice (*Oryza sativa*) [37] and sugar beet (*Beta vulgaris*) [54], *Arabidopsis* embryos [58] and soybean (*Glycine max*) seeds [57], although at lower levels than reported here (17 %, 14 %, 5 and 9 % respectively). Elevation in CHH methylation appears to accompany dedifferentiation in plant cells, though the high levels are not typically retained in plants regenerated from these tissues [58].

In plants, asymmetric CHH methylation is catalysed by heterochromatin-targeting CMT2 and by the 24 nt siRNA-guided Pol IV-RdDM pathway. In this study we found that CMT2 was more highly expressed in EC than leaf and that TEs in intergenic regions carried higher CHH methylation than those within genes (Fig. 2A). The relatively long 24 nt siRNAs were also found to be most abundant in EC (Fig. 4A), and these het-siRNA were enriched around TE features (Fig. 4B). These findings suggest both CMT2 and RdDM contribute to high CHH methylation in EC. While TE-specific CHG methylation was higher overall in EC than in leaf (Fig. 1 A), no difference was seen in the expression of CMT3, which maintains CHG methylation following DNA replication. Interestingly, TE methylation in this context was dependent on the genomic context of the TEs. The majority of genic TEs had lower methylation in EC, but TEs in intergenic regions generally had higher CHG methylation (Fig. 2B). These results suggest that in EC cultures, the context of TEs with regard to genes is an important factor in determining whether they are silenced, which led us to analyse whether transcription of TEs depends on the transcription of co-located genes. Transcript libraries obtained from EC contained approximately twice the proportion of TE-mapping reads as those from leaf tissue. The relative abundance of TE transcripts was not due to high copy numbers of a few TE families. Rather, most TE families (85 out of 122) were significantly more highly expressed in EC. Analysing only unique-mapping transcript reads revealed that the majority of DE TEs were indeed co-located with DE genes.

**Fig. 6:**
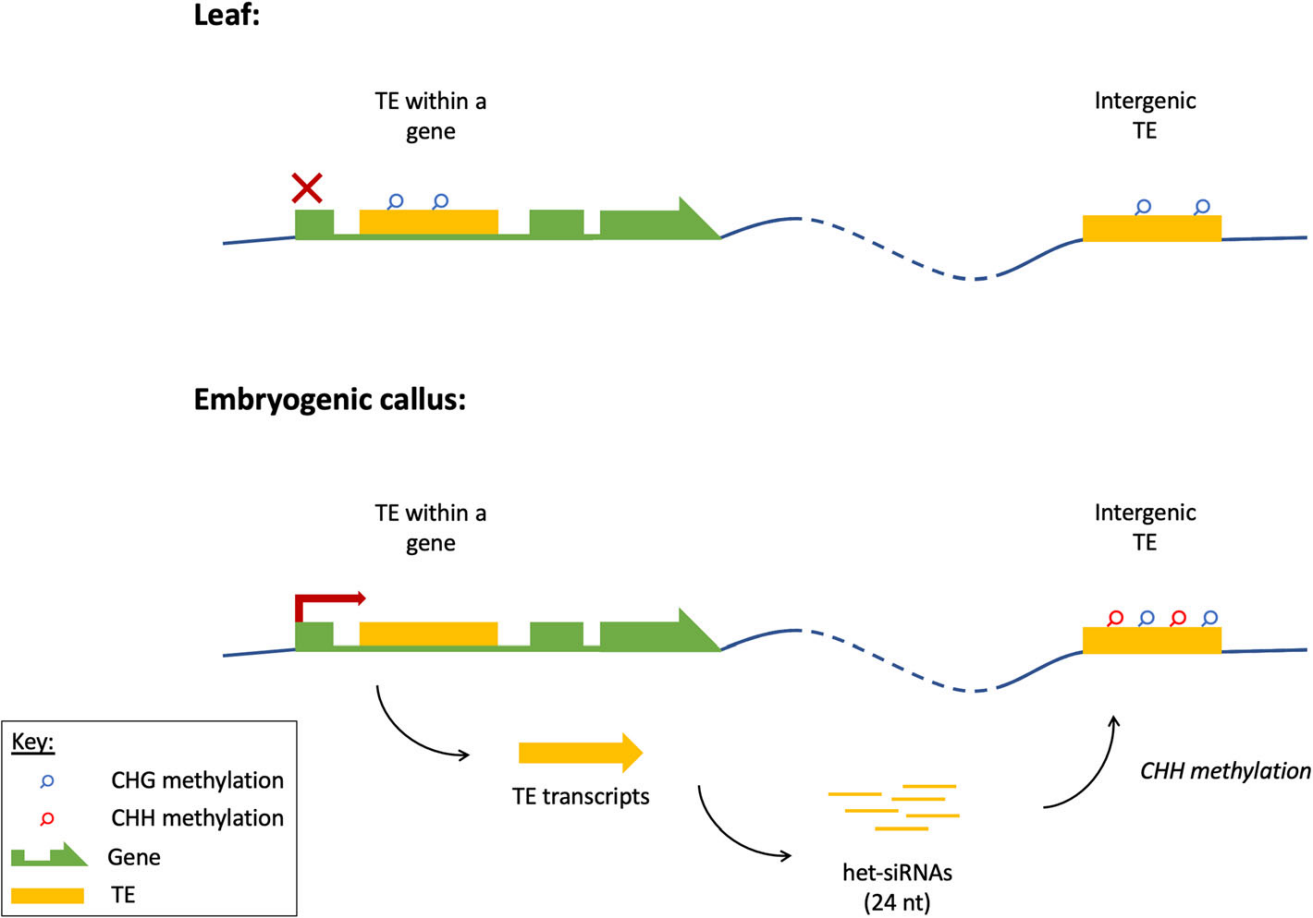
A schematic overview of TE expression and silencing in leaf and EC. In leaf tissue, TE expression is generally low, with both genic and intergenic TEs methylated in CHG context. In EC, genic TEs have reduced CHG methylation and are transcribed with the genes in which they are co-located. This triggers RdDM, in which the TE transcripts are processed into 24 nt het-siRNAs that guide *trans* silencing of repeat sequences across the genome

By integrating the three data types, we suggest a descriptive model for the epigenetic state of TEs in grapevine EC. In these cultures, a diverse set of genic TEs are co-transcribed with the genes in which they lie. This appears to be associated with a reduction in CHG methylation across these elements. The increase in TE transcript abundance triggers a response in the form of het-siRNAs with sequence-specificity for the transcribed TEs. Consequently, the genome experiences a burst of CHH methylation via Pol IV-RdDM, leading intergenic TEs across the genome to be silenced in *trans* (Fig. 6). The genic context of TEs is key to understanding the finding that TEs in EC are both more methylated and more highly transcribed than in leaves. This finding seems at odds with the general belief that a primary function of epigenetic modifications (particularly CHG methylation, in the case of plants) is to silence TEs. However as discussed above, genic TEs in EC were relatively hypomethylated, but since there are far more intergenic TE copies which are hypermethylated, the total, aggregated methylation of TEs in EC was higher than in leaf. The reason that EC has reduced CHG methylation at genic TEs is not known, but a similar observation has previously reported in the case of the *Bad Karma* allele in oil palm [31]. The cause does not appear to be a decrease in CMT3 expression. It is notable the EC cells contained lower 21–22 nt siRNA abundance than leaf, and that the enrichment of 21 nt siRNA clusters across genes that was seen in leaf was almost absent in EC.

In plants, a general increase in TE expression is known to occur during DRTS, a tissue-specific phenomenon in which genome-wide loss of epigenetic silencing is accompanied by high rates of TE transcription and transposition [59]. DRTS has been observed in differentiated tissues adjacent to rapidly-multiplying pluripotent cells, including the pollen vegetative nucleus and endosperm in *Arabidopsis* and maturing leaves in maize [60–62]. It has been proposed that one function of DRTS may be to trigger the production of 21–22 nt small RNAs that are able to migrate into adjacent germline or meristematic tissues where they reinforce TE silencing via RNAi and *RDR6*-dependent RdDM [59, 62]. Despite the similar accumulation of TE transcripts from multiple families, the situation in grapevine EC differs in certain regards to canonical examples of DRTS. In *Arabidopsis*, heterochromatin loss is caused by reduced expression of the chromatin remodeller DDM1, allowing TE transcription [61]. In contrast, grapevine EC showed comparably high expression of DDM1 and DNA methyltransferases, while 21–22 nt siRNA levels were lower than in leaf tissue. Instead, 24 nt het-siRNAs, which target TEs for asymmetric CHH methylation, were most abundant. Rather than an example of DRTS, the case of grapevine EC may instead be analogous to an embryo lacking an adjacent endosperm. In this scenario, TEs co-located with expressed genes are transcribed and, without a source from which to import secondary siRNAs to guide RNAi, their transcripts accumulate.

If EC is indeed analogous to naturally-occurring dedifferentiated tissues, it could provide a useful system in which to test current hypotheses about the evolutionary function of DRTS in plants. Most pertinent among these is the hypothesis that without secondary siRNAs imported from nearby tissue, TE transcripts in dedifferentiated cells would overwhelm the PTGS system and exceed the cells’ ability to prevent transposition. Further work is needed to establish whether the high rates of transposition events observed in totipotent plant cell cultures can directly be attributed to a lack of imported secondary siRNAs. In this study, the transcription of TEs in EC was associated with a burst of *de novo* CHH methylation. Similarly, it remains to be seen whether this strand-specific methylation results in stable epigenetic changes in plants regenerated from these cultures, and if so, whether new epialleles affect the expression of nearby genes. In other species, increased CHH methylation is not typically seen in regenerated plants [58]. However, plants regenerated from tissue culture do frequently (though not consistently) demonstrate epigenetic variation, including in grapevine [28, 30, 39].

## Conclusions

In grapevine, transcription of almost all known TE superfamilies is higher in dedifferentiated EC cells of grapevine than in leaf tissue. This appears to be due to a the loss of CHG methylation across TEs within genes and the transcription of those genes. The accumulating TE transcripts are accompanied by a genome-wide epigenetic response involving an increase in het-siRNAs and *de novo* methylation of TE DNA in *trans*. It remains to be seen whether the burst of TE transcription and associated epigenetic response persists as somaclonal genetic or epigenetic variation in vines regenerated from these cultures.

These results add insight into the epigenetic regulation of transposition, particularly in embryogenic cell cultures. Understanding these factors is important given their use in studying embryogenesis and for crop improvement. Better control of transposition and epiallele formation will improve outcomes for producing new somaclonal variation, gene discovery and targeted gene editing, homogenous propagation of elite genotypes, and managing chimerism in plants regenerated from cell culture.

## Methods

### TE annotation

Transposable elements in the *V. vinifera* PN40024 12X reference genome [63] were annotated with RepeatMasker (v4.1.0; RRID:SCR_012954) [64]. Query sequences included *V. vinifera* repeat sequences from Repbase (RRID:SCR_021169) [65] supplemented with *V. vinifera* repeat sequences collected from published literature [11]. TE annotations were assigned a custom nomenclature that included class, superfamily, family and locus, for downstream analysis and sorting. Feature overlaps were identified by comparing annotated TEs with gene features from the *V. vinifera* V2.1 structural annotation [66], using the intersect function of the BEDtools toolkit (v2.29.2; RRID:SCR_006646) [67].

### Plant material

Anther tissue was selected for EC initiation, due to the broad applicability of this technique across multiple grapevine varieties. Embryogenic cell cultures were established using the protocol of Perrin and colleagues [68] from anthers of immature inflorescences harvested from *V. vinifera* cv ‘Chardonnay’, grown at the Lincoln University research vineyard (Canterbury, New Zealand). Established cultures were grown on solid C_1_P media at 26 ± 1 ˚C in the dark [69]. After three months, healthy white EC masses were transferred to fresh C_1_P media, leaving behind brown dark brown callus and anthers that showed no callus formation. Four months after initial callus formation, samples of these proliferating EC cultures from three Petri dishes were pooled per sample and harvested directly into liquid N_2_. For leaf tissue samples, young leaves (< 30 mm in diameter) were selected, which yield high quality DNA and RNA in grapevine. Samples were collected from the same vines described above (five leaves per sample) and harvested directly into liquid N_2_. Samples were stored at -80˚C until used for DNA and RNA purification.

### DNA methylation analysis

Genomic DNA was extracted from 50 mg aliquots of young leaf and embryogenic callus samples ground in liquid N_2_, using the NucleoMag® Plant DNA kit (Macherey-Nagel GmbH) according to the manufacturer’s instructions. Isolated DNA was stabilised using DNAstable (Biomātrica) and shipped to Macrogen (Seoul, Rep. of Korea) for library preparation and sequencing. Methylation-specific sequencing libraries were prepared using the TruSeq DNA Methylation kit (Illumina, Inc.) according to the manufacturer’s specifications. Leaf and embryogenic callus libraries were multiplexed and sequenced on two lanes of a HiSeq 2000 flow cell using 100 bp paired-end sequencing.

Read pairs were mapped to the 12X PN40024 grapevine reference genome using BS-Seeker2 (v2.1.8; RRID:SCR_020948) [70] with the parameters ‘-X 1000 -m 0.04’. Methylation calling and global methylation statistics were generated with the ‘bam2cgmap’ tool from the cgmaptools programme package [71]. Methylation across features and methylation per feature were calculated using the ‘mfg’ and ‘mtr’ functions from the same package, respectively. Only features which had a methylation effective coverage greater than 20 in both samples were included in the analysis of methylation across features. Data visualisation was performed in R (v4.0.2; RRID:SCR_001905). Bisulphite conversion efficiency was calculated by calculating methylation levels of all reads mapping to the non-methylated chloroplast genome (NCBI reference sequence: NC_007957.1).

### Transcriptome analysis

RNA was purified from triplicate EC and leaf tissue samples using the Spectrum™ Plant Total RNA kit (Sigma-Aldrich). Isolated RNA was quality checked using an Bioanalyzer 2100 with the RNA 6000 nano kit (Agilent Technologies, Inc.) according to manufacturer’s instructions and OD_260/280_ ratios determined with a DS-11 spectrophotometer (DeNovix, Inc.). Quantification of RNA samples was done using a Qubit fluorometer (Thermo Fisher Scientific) and the samples were stabilised using RNAStable (Biomātrica, Inc.) prior to shipping to Macrogen for sequencing.

Library synthesis and sequencing was carried out by Macrogen (Seoul, Rep. of Korea). Briefly, libraries were prepared using a TruSeq Stranded Total RNA kit (Illumina, Inc.) with ribosomal RNA removed using the Ribo-Zero Plant (leaf) kit (Illumina, Inc.). Prepared libraries were multiplexed and sequenced on a single lane of a HiSeq2000 DNA sequencer (Illumina, Inc.) to yield 100 bp paired-end reads.

Adapter clipping and quality trimming of reads was carried out using FastQC (v0.11.9; RRID:SCR_014583) [72] and fastq-mcf [73] with default parameters except for a minimum read length of 36. Reads were mapped to tRNA and rRNA references sequences downloaded from the rfam database (v12.0; RRID:SCR_007891) [74] and *V. Vinifera* tRNA sequences downloaded from GtRNAdb (v18.1; RRID:SCR_006939) [75] using HISAT2 (v2.2.1; RRID:SCR_015530) [76]. Reads that mapped to tRNA and rRNA sequences were excluded. Reads were then mapped to the *V. vinifera* PN40024 12X reference genome [63] using HISAT2 with the parameter ‘-k 100’ to allow multi-mapping. Gene and TE transcripts were counted simultaneously with TEtranscripts (v2.2.1) [77], using the V2.1 gene and TE annotation tracks described above.

Read counts for two genes demonstrating evidence of genome over-assembly (VIT_213s0019g0263 and VIT_207s0031g03000) were removed from raw count tables prior differential expression (DE) analysis using DEseq2 (v1.28.1; RRID:SCR_015687) [78]. Genes showing DE (Benjamini-Hochberg adjusted *p*-value < 0.05) and a 2x change between tissues were considered to be differentially expressed genes (DEGs). Gene ontology (GO) terms associated with DEGs were retrieved from the CRIBI database (http://genomes.cribi.unipd.it/DATA/V2/annotation/bl2go.annot.txt), and enrichment analysis was performed using the fatiGO algorithm of the Babelomics software package (v5; RRID:SCR_002969) [79]. GO terms with FDR-adjusted *p*-values < 0.01 were then passed to REViGO (RRID:SCR_005825) for grouping [80].

To compare the expression of TEs with co-located genes, only reads uniquely mapping to the genome were used. Reads mapping to exons and TEs (exons excluded) were counted separately using featureCounts (v2.0.0; RRID:SCR_012919) [81]. Count tables for both feature types were concatenated and analysed for DE using DEseq2 per Macchietto et al. [82].

All libraries were also mapped to the *V. Vinifera* cv. ‘Chardonnay’ reference genome [83] Although mapping reads to the Chardonnay reference resulted in a higher proportion of mapped reads, the majority of these were not properly paired and would therefore not be included in the downstream analysis pipeline (Suppl. Table S5). Instead, alignments to the more commonly used PN40024 reference genome, which showed high mapping rates as well as high proportions of properly paired reads, were used for further analysis.

### Small RNA analysis

Small RNA (< 200 nt) was purified from 50 mg each of embryogenic cell cultures and young leaf tissue described above, using the Plant microRNA Purification Kit (Norgen BioTek Corp.) according to manufacturer’s instructions. RNA samples were sent to New Zealand Genomics Ltd. (Auckland, New Zealand), where a 18–35 nt fraction of the each RNA sample was isolated by polyacrylamide gel electrophoresis and sequencing libraries were prepared using the TruSeq Small RNA library Preparation kit (Illumina, Inc.). The two libraries were sequenced on a single flow cell on a MiSeq DNA sequencer (Illumina, Inc.) using a 50 bp single-end sequencing protocol.

Adapter trimming and size filtering of sequence reads was performed in the UEA small RNA Workbench (v4.5; RRID:SCR_020947) [84]. Reads that mapped to *V. vinifera* tRNA and rRNA sequences, as described above, were removed using PatMaN (v1.2.2; RRID:SCR_011821) [85]. Identification of miRNA and their removal has been described elsewhere [42]. After removal of miRNAs, small RNAs were mapped to the reference genome using ShortStack (v3.8.5; RRID:SCR_010834) [86], which selects unique mapping sites for multi-mapping reads based on the location of unique-mapping reads. ShortStack was also used to perform siRNA cluster discovery and assign DicerCalls, based on the predominant fragment size for each cluster. Enrichment of 20 to 24 nt cluster sizes across three genomic feature types: genes, promoters (defined as regions 2 kb upstream of genes) and TEs, was assessed using Genomic Association Tester (v1.3.6; RRID:SCR_020949) [87].

## Supplementary Information

**Additional file 1: Figure S1. **TE abundance in the Vitis vinifera genome. A: Counts of annotated TEs per superfamily. B: Total coverage of the reference genome for each TE superfamily (total coverage by exons is plotted for comparison).

**Additional file 2: Figure S2.** Gene Ontology terms enriched among DE genes highly expressed in leaf tissue relative to EC. Enriched terms were grouped by similarity using REVIGO.

**Additional file 3: Table S1. **Sequencing libraries used in this study.

**Additional file 4: Table S2.** Genes differentially expressed between EC and leaf.

**Additional file 5: Table S3.** TE families differentially expressed between EC and leaf.

**Additional file 6: Table S4. **Sequence reads remaining after successive filtering steps for small RNA libraries.

**Additional file 7: Table S5.** Mapping statistics for read libraries.

## Data Availability

The data generated in this study have been deposited in Gene Expression Omnibus under the accession number GSE164586, except for the TE annotation track, which is available at DOI: 10.6084/m9.figshare.14709816. The *V. vinifera* PN40024 12X reference genome used is available under the GenBank assembly accession number GCA_000003745.2. and the *V. vinifera* chloroplast sequence used is available under the GenBank accession number NC_007957.1.

## Abbreviations

DE: Differentially expressed
DRTS: Developmental relaxation of TE silencing
dsRNA: Double-stranded RNA
EC: Embryogenic callus
FDR: False discovery rate
GAT: Genomic Association Tester
GO: Gene ontology
het-siRNA: Heterochromatic small interfering RNA
LINEs: Long interspersed nuclear elements
miRNA: micro RNA
MULE: Mutator-like element
PTGS: Post-transcriptional gene silencing
RdDM: RNA-directed DNA methylation
RNAi: RNA interference
RPM: Reads per million
siRNA: Small interfering RNA
sRNA: Small RNA
TE: Transposable element
TGS: Transcriptional gene silencing
