# Supplementary figures and images for: Elevated transcription of transposable elements is accompanied by het-siRNA-driven de novo DNA methylation in grapevine embryogenic callus
Source: BMC Genomics. 2021 Sep 20;22:676. doi: 10.1186/s12864-021-07973-9 (PMC8454084; doi:10.1186/s12864-021-07973-9)

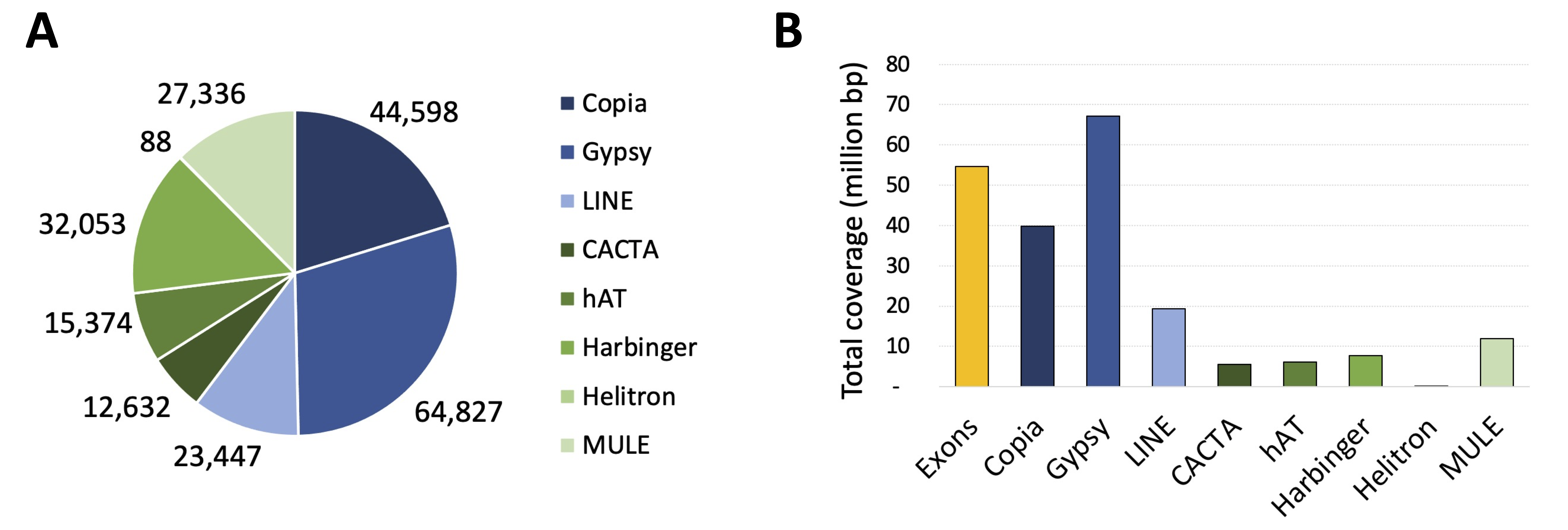

Supplement: Supplementary file 1 — Additional file 1: Figure S1. TE abundance in the Vitis vinifera genome. A: Counts of annotated TEs per superfamily. B: Total coverage of the reference genome for each TE superfamily (total coverage by exons is plotted for comparison). [file 12864_2021_7973_MOESM1_ESM.png]

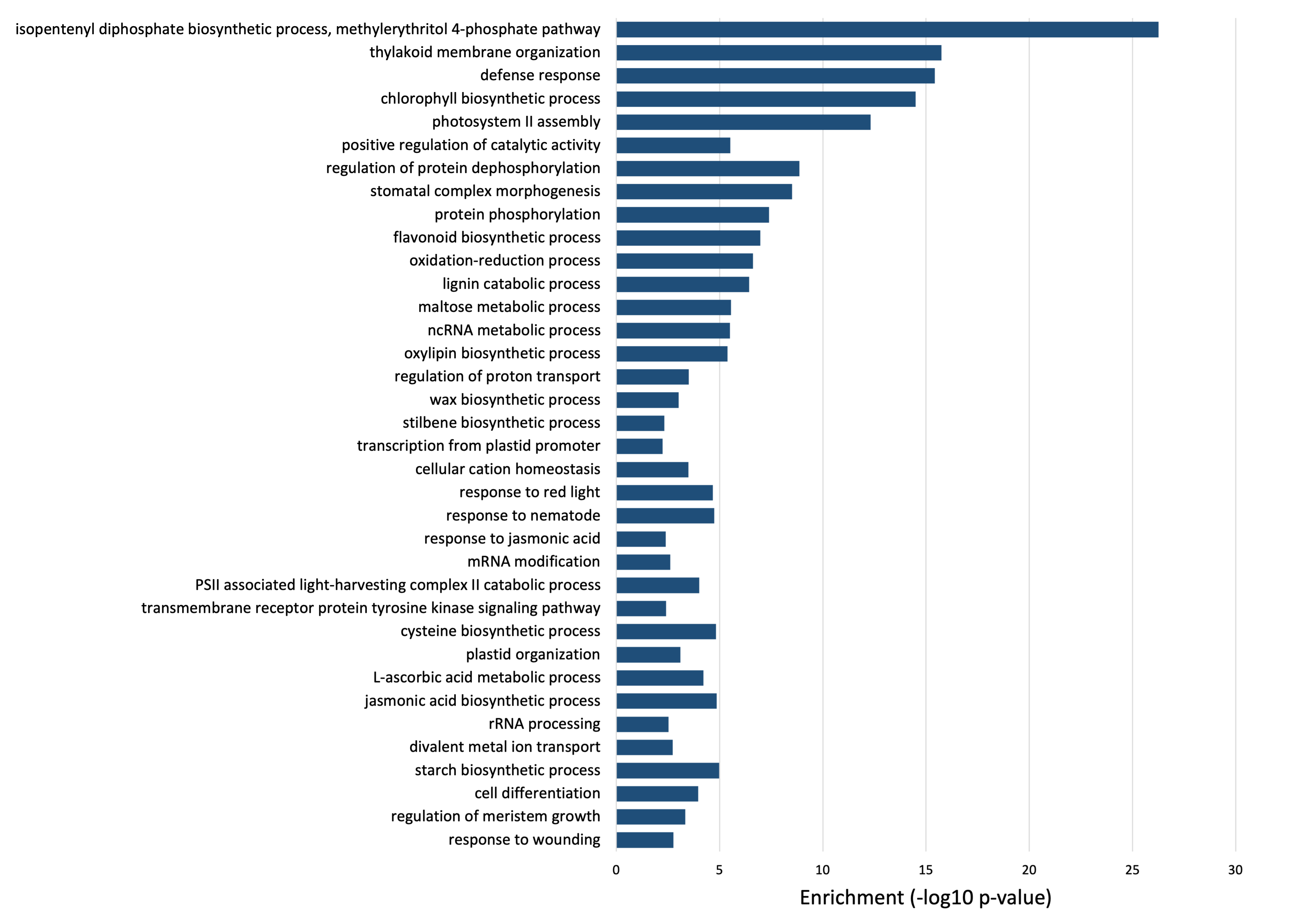

Supplement: Supplementary file 2 — Additional file 2: Figure S2. Gene Ontology terms enriched among DE genes highly expressed in leaf tissue relative to EC. Enriched terms were grouped by similarity using REVIGO. [file 12864_2021_7973_MOESM2_ESM.png]
