# Supplementary material for: Elevated transcription of transposable elements is accompanied by het-siRNA-driven de novo DNA methylation in grapevine embryogenic callus
Source: BMC Genomics. 2021 Sep 20;22:676. doi: 10.1186/s12864-021-07973-9 (PMC8454084; doi:10.1186/s12864-021-07973-9)
Supplement: Supplementary file 3 — Additional file 3: Table S1. Sequencing libraries used in this study. [file 12864_2021_7973_MOESM3_ESM.docx]

**Table S1:**

| **Sample** | **Tissue type** | **Library type** | **Sequencing strategy** | **SRA number** | **Reads (pairs)** | **mean read coverage** |
| --- | --- | --- | --- | --- | --- | --- |
| WGBS_leaf | Leaf | bisulphite-converted DNA | 100bp PE | GSM5014396 | 145,014,795 | 36.43 |
| WGBS_callus | Embryogenic callus | bisulphite-converted DNA | 100bp PE | GSM5014397 | 151,656,744 | 32.35 |
| RNA-seq_Leaf1 | Leaf | ribosome-depleted RNA | 100bp PE | GSM5014398 | 39,362,786 | - |
| RNA-seq_Leaf2 | Leaf | ribosome-depleted RNA | 100bp PE | GSM5014399 | 36,176,858 | - |
| RNA-seq_Leaf3 | Leaf | ribosome-depleted RNA | 100bp PE | GSM5014400 | 36,693,590 | - |
| RNA-seq_EC1 | Embryogenic callus | ribosome-depleted RNA | 100bp PE | GSM5014401 | 36,841,252 | - |
| RNA-seq_EC2 | Embryogenic callus | ribosome-depleted RNA | 100bp PE | GSM5014402 | 35,358,849 | - |
| RNA-seq_EC3 | Embryogenic callus | ribosome-depleted RNA | 100bp PE | GSM5014403 | 38,294,117 | - |
| Leaf_smallRNA | Leaf | small RNA | 50bp SE | GSM5014404 | 7,886,332 | - |
| EC_smallRNA | Embryogenic callus | small RNA | 50bp SE | GSM5014405 | 7,010,188 | - |
