# Supplementary material for: Elevated transcription of transposable elements is accompanied by het-siRNA-driven de novo DNA methylation in grapevine embryogenic callus
Source: BMC Genomics. 2021 Sep 20;22:676. doi: 10.1186/s12864-021-07973-9 (PMC8454084; doi:10.1186/s12864-021-07973-9)
Supplement: Supplementary file 6 — Additional file 6: Table S4. Sequence reads remaining after successive filtering steps for small RNA libraries. [file 12864_2021_7973_MOESM6_ESM.docx]

**Table S4:**

|  | **Leaf** | |  | **EC** | |
| --- | --- | --- | --- | --- | --- |
| **Library filtering step** | **Total reads** | **Distinct reads** |  | **Total reads** | **Distinct reads** |
| Total read sequences | 7,886,332 (100%) | 1,238,982 (100%) |  | 7,010,188 (100%) | 2,294,748 (100%) |
| 3' adaptor removal & quality filtering | 7,633,928 (97%) | 1,009,121 (81%) |  | 6,780,664 (97%) | 1,977,933 (86%) |
| Filtered by length | 6,738,881 (85%) | 936,553 (76%) |  | 4,842,717 (69%) | 1,821,213 (79%) |
| tRNA/rRNA/snoRNA removed | 4,859,237 (62%) | 898,097 (72%) |  | 3,439,527 (49%) | 1,776,990 (77%) |
| miRNA removed | 2,818,653 (36%) | 898,012 (72%) |  | 3,411,631 (49%) | 1,776,928 (77%) |
| Mapped to genome | 1,587,096 (20%) | 515,541 (42%) |  | 2,117,178 (30%) | 1,098,195 (48%) |
