# Supplementary material for: Elevated transcription of transposable elements is accompanied by het-siRNA-driven de novo DNA methylation in grapevine embryogenic callus
Source: BMC Genomics. 2021 Sep 20;22:676. doi: 10.1186/s12864-021-07973-9 (PMC8454084; doi:10.1186/s12864-021-07973-9)
Supplement: Supplementary file 7 — Additional file 7: Table S5. Mapping statistics for read libraries. [file 12864_2021_7973_MOESM7_ESM.docx]

**Table S5:**

| ***A: Mapping statistics for mRNA reads*** | | | |  |
| --- | --- | --- | --- | --- |
| **Reference genome** | **PN40024** | | **Chardonnay** | |
| **Library** | **Mapping** | **Properly paired** | **Mapping** | **Properly paired** |
| **EC1** | 87.7% | 81.7% | 91.2% | 11.8% |
| **EC2** | 88.0% | 83.0% | 91.4% | 11.9% |
| **EC3** | 87.8% | 82.7% | 92.2% | 11.8% |
| **Leaf1** | 86.4% | 80.4% | 92.4% | 12.0% |
| **Leaf2** | 86.5% | 80.8% | 92.1% | 12.7% |
| **Leaf3** | 80.5% | 72.4% | 97.0% | 10.7% |

| ***B: Mapping statistics for bis-seq reads*** | |  |
| --- | --- | --- |
| **Reference genome** | **PN40024** | **Chardonnay** |
| **Library** | **Mapped pairs** | **Mapped pairs** |
| **EC** | 64.8% | 70.8% |
| **Leaf** | 69.2% | 67.7% |

| ***C: Mapping statisticss for siRNA reads*** | | |  | |  | |  | | |
| --- | --- | --- | --- | --- | --- | --- | --- | --- | --- |
| **Reference genome** | **PN40024** | | | **Chardonnay** | | | |  |  |
| **Library** | **Uniquely mapped** | **Multi mapped** | | **Uniquely mapped** | | **Multi mapped** | | |  |
| **EC** | 38.8% | 38.2% | | 31.6% | | 49.70% | | |  |
| **Leaf** | 36.5% | 33.6% | | 27.3% | | 46.60% | | |  |
